# Dr. Albert E. Warren

**Published:** 1905-10

**Authors:** 


DR. ALBERT E. WARREN, of Youngstown, O., a graduate of the University of Buffalo, Medical Department, 1891, committed suicide by shooting at his father's residence in this city, October 6, 1905, aged 36 years. Dr Warren had suffered ill health for some time having contracted sepsis at an operation two years ago.

